# Effects of tannic acid on growth performance, relative organ weight, antioxidative status, and intestinal histomorphology in broilers exposed to aflatoxin B_1_

**DOI:** 10.3389/fvets.2022.1037046

**Published:** 2022-10-21

**Authors:** Yu Xi, Jing Chen, Shuangshuang Guo, Sitian Wang, Zhipeng Liu, Liyun Zheng, Ya Qi, Pengtao Xu, Lanlan Li, Zhengfan Zhang, Binying Ding

**Affiliations:** Hubei Key Laboratory of Animal Nutrition and Feed Science, Wuhan Polytechnic University, Wuhan, China

**Keywords:** Aflatoxin B1, antioxidant capacity, broiler, growth performance, intestinal health, tannic acid

## Abstract

A total of 480 one-day-old AA broiler chicks were randomly allocated to one of four treatments in a 2 × 2 factorial to investigate the effects of tannic acid (TA) on growth performance, relative organ weight, antioxidant capacity, and intestinal health in broilers dietary exposed to aflatoxin B_1_ (AFB_1_). Treatments were as follows: (1) CON, control diet; (2) TA, CON + 250 mg/kg TA; (3) AFB_1_, CON + 500 μg/kg AFB_1_; and (4) TA+AFB_1_, CON + 250 mg/kg TA + 500 μg/kg AFB_1_. There were 10 replicate pens with 12 broilers per replicate. Dietary AFB_1_ challenge increased the feed conversion ratio during days 1 to 21 (*P* < 0.05). The TA in the diet did not show significant effects on the growth performance of broilers during the whole experiment period (*P* > 0.05). The liver and kidney relative weight was increased in the AF challenge groups compared with the CON (*P* < 0.05). The addition of TA could alleviate the relative weight increase of liver and kidney caused by AFB_1_ (*P* < 0.05). Broilers fed the AFB_1_ diets had lower activity of glutathione peroxidase, catalase, total superoxide dismutase, S-transferase, and total antioxidant capacity in plasma, liver and jejunum, and greater malondialdehyde content (*P* < 0.05). Dietary supplemented with 250 mg/kg TA increased the activities of antioxidative enzymes, and decreased malondialdehyde content (*P* < 0.05). In addition, AFB_1_ significantly reduced the villus height and crypt depth ratio in the ileum on day 42 (*P* < 0.05). In conclusion, supplementation with 250 mg/kg TA could partially protect the antioxidant capacity and prevent the enlargement of liver in broilers dietary challenged with 500 μg/kg AFB_1_.

## Introduction

Aflatoxins are mainly produced by *Aspergillus flavus* and *Aspergillus parasiticus*, and widely exist in food and feed that are frequently caused health and economic problems in many countries (1). Among the 18 types of aflatoxin derivatives, aflatoxin B_1_ (AFB_1_) is the most common and toxic in the poultry feed industry (2). Poultry is extremely sensitive to AFB_1_, and long-term exposure to AFB_1_ may cause growth retardation, immunosuppression, hepatotoxic, and even death (3–5). Oxidative stress has been reported to play a significant role in the toxicity mechanism caused by AFB_1_ (6, 7). FDA (8) refines the maximum concentration of aflatoxin in poultry is 100 μg/kg of feed, whereas 500 μg/kg can be a practical testing concentration in feedstuff in the USA.

Chinese gallnut tannic acid (TA) belongs to the hydrolyzed tannin family, and is a polyphenolic compound of high molecular weight (500–3,000 Da), which can remove free radicals and prevent lipid oxidation (9). Because of the polyphenolic hydroxyl structure, the TA has various biological activities, such as antimicrobial, anti-inflammatory, anticancer, and immunomodulatory effects (10–12). Moreover, studies have shown that dietary supplementation with antioxidants, including plant extracts and tannins can protect broilers from AFB_1_-induced toxicity by enhancing the antioxidant capacity and immunity (6, 13–16). Nevertheless, it remains unclear whether dietary supplementation with TA could alleviate acute aflatoxicosis by improving the antioxidant capacity of broilers fed AFB_1_ contaminated diets.

Therefore, the aim of this study was to determine the effects of the TA on growth performance, antioxidative status, and intestinal histomorphology of broilers exposed to feed contaminated with 500 μg/kg AFB_1_.

## Materials and methods

All animal procedures used in this study were performed in the experimental farm of Wuhan Polytechnic University, and were approved by the Institutional Animal Care and Use Committee of Wuhan Polytechnic University (Number: 20161121).

### AFB_1_ and TA

The AFB_1_ (purity ≥98%, HPLC) was produced from *Aspergillus flavus* provided by Qingdao Pribolab Biological Engineering Company Limited (Shandong, China), and the AFB_1_ concentration in the feed was designed to 500 μg/kg in AFB_1_ treatments. Dietary AFB_1_ concentrations were confirmed by analysis (17). Briefly, feed samples were extracted with acetonitrile:water (86:14), and an aliquot of the extract was passed through a puriTox TC-M160 cleanup column (Trilogy Analytical Laboratory Inc., Washington, MO, USA) and suitably diluted with water before analysis using HPLC with Kobra cell postcolumn derivatization with fluorescence detection at 365 nm excitation and 440 nm emission.

The hydrolysable TA was extracted from Chinese gallnut by the Wufeng Chicheng Biotechnology Company Limited (Yichang, China), which contained ≥80% tannin, crude fiber <2.00%, ash <2.50%, and moisture <8.00%.

### Dietary treatments and animal management

A 2 × 2 factorial complete randomized block design was employed and 480 one-day-old sex-mixed AA broilers were randomly assigned to 4 treatment groups, each with 10 replicates of 12 birds per pen. Experimental diets were as follows: (1) CON, basal diet; (2) TA, CON + 250 mg/kg A; (3) AFB_1_, CON + 500 μg/kg TA; and (4) TA+AFB_1_, CON + 250 mg/kg TA + 500 μg/kg AFB_1_. The basal diet was formulated to meet or exceed the nutrient requirements of AA broilers. Diets were fed in 2 phases: phase 1 (from days 1 to 21) and phase 2 (from days 22 to 42). The composition and nutrient levels of the basal diets are presented in Table 1.

**Table 1:** Composition of experimental diets (as-fed basal).

**Ingredients (%)**	**Days 1–21**	**Days 22–42**
Corn	51.45	51.49
Soybean meal	40.73	37.40
Soybean oil	3.36	7.18
Dicalcium phosphate	1.92	1.64
Limestone	1.16	1.06
Trace mineral premix^a^	0.20	0.20
Vitamin premix^b^	0.04	0.03
Sodium chloride	0.35	0.31
l-Lysine (99%)	0.28	0.22
dl-methionine (98%)	0.26	0.32
Choline chloride	0.25	0.25
**Calculated composition**
ME (MJ/kg)	12.55	13.18
**Analyzed composition**
Crude protein (%)	21.50	20.50
Lys (%)	1.30	1.20
Met + Cys (%)	0.90	0.70
Thr (%)	0.82	0.74
Calcium (%)	1.00	0.90
Available phosphorus (%)	0.45	0.40

^a^ Provided per kg of complete diet: 10 mg Mn (MnSO_4_), 80 mg Zn (ZnSO_4_), 5 mg Cu (CuSO_4_), 0.5 mg I (Ca(IO_3_)_2_), and 0.3 mg Se (Na_2_SeO_3_).

^b^ Provided per kg of complete diet: 10,000 IU vitamin A (transretinyl acetate), 3,000 IU vitamin D_3_ (cholecalciferol), 30 IU vitamin E (all-rac-α-tocopherol acetate), 2.4 mg menadione, 6.0 mg riboflavin, 2.5 mg pyridoxine HCl, 13 mg calcium pantothenate, 23.5 mg niacin, and 0.04 mg biotin.

All broiler chicks were reared in stainless steel pens (1.4 m × 1.4 m) in an environmentally controlled room at the Animal Research Center of Wuhan Polytechnic University and given *ad libitum* access to diets and water throughout the study. The room temperature was maintained at 33 ± 2°C for the first week and then gradually decreased to 24°C until the end of the experiment, and broilers were maintained on a 23 h constant light and 1 h darkness every day throughout the whole trial.

### Growth performance

Broilers and feed were weighed on the beginning, days 21 and 42 of the trial, and calculated the average daily gain (ADG), average daily feed intake (ADFI), and feed conversion ratio (FCR).

### Sample collection

On days 21 and 42, two broilers from each replicate (20 broilers per group) were randomly selected and blood samples were aseptically collected from the wing vein into vacuum blood vessels. Plasma was obtained by centrifuging (3,000 × g for 15 min at 4°C) the whole blood and stored at −20°C for the assay of antioxidative parameters.

Then, the same broilers were weighed individually and euthanized by cervical dislocation. The liver, spleen, bursa of Fabricius, thymus, and kidney were removed cleaned of the adhering tissue by trained personnel and weighed. Relative organ weights were calculated as follows: Relative weight = (Organ weight)/(Final body weight) × 1,000. The small intestine was removed and gently cleaned with ice-cold saline. Intestinal segments (1–2 cm) taken from the mid-region of the duodenum, jejunum, and ileum were immediately fixed in 4% paraformaldehyde for the examination of morphological parameters. Additionally, the portion of liver and jejunum were sampled and stored at −80°C for analysis of antioxidant status.

### Antioxidative status

Approximately 1 g of liver or jejunum was homogenized in 10 mL of ice-cold saline and centrifuged at 2,500 × g, 4°C for 10 min. The supernatants were collected for further analysis. The activities of glutathione peroxidase (GSH-Px), total superoxide dismutase (T-SOD), total antioxidant capacity (T-AOC), glutathione S-transferase (GST), catalase (CAT), and the content of malondialdehyde (MDA) in the plasma and supernatants were measured using purchased assay kits (Nanjing Jiancheng Bioengineering Institute, Nanjing, China), according to the instructions of the manufacturer (18).

### Intestinal histomorphology

The intestinal histomorphology was measured as described by Guo et al. (19). Briefly, the fixed intestinal segments were embedded in paraffin. Consecutive sections (5 μm) were stained with hematoxylin and eosin and were observed for histomorphological examination. The measurements were performed with an Olympus optical microscope using ProgRes CapturePro software (Jenoptik, Jena, Germany). The villus height and crypt depth were measured from 10 randomly selected villi and associated crypts on each section at 40 × magnification. Villus height was measured from the tip of the villus to the crypt opening and crypt depth was measured from the base of the crypt to the level of the crypt opening. The villus height to crypt depth ratio (V/C) was then calculated from these measurements.

### Statistical analyses

All experiment data were analyzed by a two-way ANOVA analysis using the GLM procedure of SPSS 26.0 software. In cases where the differences were significant, the means were compared by Duncan's multiple range test. The results are shown as mean and the standard error of mean (SEM). Significance was considered at *P* < 0.05, and 0.05 ≤ *P* < 0.10 was considered to have a trend of difference.

## Results

### Dietary analyses of AFB_1_

Biochemical tests indicated that the CON and TA diets were negative for AFB1 throughout the experiment. The analyzed concentration of AFB1 in AFB1 and AFB1+TA diets were 505.9 vs. 503.2 μg/kg during days 1 to 21, and 520.3 vs. 521.3 μg/kg during days 22–42, respectively.

### Growth performance

As shown in Table 2, AFB_1_ challenge increased the FCR during days 1–21 (*P* < 0.05). The addition of TA in the diet did not show significant effects on the ADG, ADFI, and FCR of broilers during the whole experiment period (*P* > 0.05). No interaction effect was observed between AFB_1_ and TA on the growth performance (*P* > 0.05).

**Table 2:** Effects of tannic acid on growth performance of broilers challenged with AFB${}_{1}^{\text{a}}$.

**Items**	**CON**	**TA**	**AFB_1_**	**TA + AFB_1_**	**SEM**	***P-*** **value**		
						**AFB_1_**	**TA**	**AFB_1_ × TA**
**Days 1–21**								
ADG (g)	32.92	32.76	33.41	32.30	0.23	0.968	0.195	0.325
ADFI (g)	46.34	46.20	47.57	46.55	0.31	0.227	0.371	0.497
FCR	1.41	1.41	1.42	1.44	0.01	0.022	0.322	0.444
**Days 22–42**								
ADG (g)	63.63	61.89	62.27	61.19	0.68	0.474	0.328	0.817
ADFI (g)	114.11	112.70	114.67	112.12	1.05	0.997	0.387	0.802
FCR	1.80	1.82	1.84	1.83	0.02	0.454	0.801	0.637
**Days 1–42**								
ADG (g)	49.06	47.68	48.72	47.51	0.42	0.774	0.153	0.923
ADFI (g)	81.33	79.59	81.30	80.44	0.60	0.751	0.323	0.733
FCR	1.66	1.67	1.67	1.69	0.01	0.475	0.460	0.757

^a^ Each mean represents 10 replications with 12 broilers per replication. CON, control diet; AFB_1_, 500 μg/kg aflatoxin B_1_ of feed; TA, 250 mg/kg tannic acid of feed; TA + AFB_1_, 250 mg/kg TA + 500 μg/kg AFB_1_.

### Relative organ weight

As shown in Table 3, on days 21 and 42, AFB_1_ and TA exhibited significant interactive effects on the relative weight of the liver and kidney in broilers (*P* < 0.05). The liver and kidney relative weight was increased in the AFB_1_ treatments compared with the CON (*P* < 0.05), while supplementation with TA into AFB_1_ contaminated diet decreased liver and kidney relative weight (*P* < 0.05). The relative weights of the spleen, bursa of Fabricius, and thymus were unaffected by AFB_1_ challenge and TA treatment on days 21 and 42 (*P* > 0.05).

**Table 3:** Effects of tannic acid on relative organ weight of broilers challenged with AFB${}_{1}^{\text{a}}$.

**Items (g/kg)**	**CON**	**TA**	**AFB_1_**	**TA + AFB_1_**	**SEM**	***P-*** **value**		
						**AFB_1_**	**TA**	**AFB_1_ × TA**
**Day 21**
Liver	20.00^b^	20.54^b^	23.67^a^	20.90^b^	0.28	<0.001	0.002	<0.001
Spleen	0.78	0.76	0.92	0.84	0.03	0.049	0.356	0.608
Bursa of Fabricius	2.89	2.93	3.10	2.98	0.07	0.391	0.781	0.596
Thymus	3.51	3.70	3.38	3.45	0.11	0.394	0.553	0.795
Kidney	7.75^b^	7.98^b^	9.27^a^	7.96^b^	0.13	<0.001	<0.001	<0.001
**Day 42**
Liver	18.20^b^	18.41^b^	22.18^a^	18.91^a^	0.33	<0.001	0.001	<0.001
Spleen	0.93	1.18	1.22	1.09	0.06	0.376	0.592	0.117
Bursa of Fabricius	2.39	2.26	2.31	2.47	0.11	0.781	0.955	0.529
Thymus	3.06	3.46	3.44	3.27	0.13	0.716	0.670	0.302
Kidney	5.78^b^	5.53^b^	7.23^a^	5.65^b^	0.13	<0.001	<0.001	<0.001

^a^ Each mean represents 10 replications with 2 broilers per replication. CON, control diet; AFB_1_, 500 μg/kg aflatoxin B_1_ of feed; TA, 250 mg/kg tannic acid of feed; TA + AFB_1_, 250 mg/kg TA + 500 μg/kg AFB_1_.

^a,b,c^Means in the same row with no common superscripts differ significantly (*P* < 0.05).

### Intestinal histomorphology

As presented in Table 4, on day 42, AFB_1_ challenge reduced the villus height and crypt depth ratio in the ileum (*P* < 0.05). The ileal villus height tended to decrease (*P* = 0.079), and the crypt depth of the jejunum tended to increase (*P* = 0.082) in AFB_1_ treatments compared with non-contaminated diets. The TA did not show significant effects on the intestinal histomorphology of broilers (*P* > 0.05). However, the villus height (*P* = 0.059) and villus height/crypt depth (*P* = 0.052) ratio were tended to increase in TA treatments. No interaction was found between AFB_1_ and TA in intestinal histomorphology (*P* > 0.05).

**Table 4:** Effects of tannic acid on intestinal histomorphology of broilers challenged with AFB${}_{1}^{\text{a}}$.

**Items^b^**		**CON**	**TA**	**AFB_1_**	**TA + AFB_1_**	**SEM**	***P-*** **value**		
							**AFB_1_**	**TA**	**AFB_1_ × TA**
**Day 21**
Duodenum	VH (μm)	1144.03	1212.71	1014.64	1133.42	32.65	0.119	0.159	0.700
	CD (μm)	89.57	92.50	86.41	86.29	2.94	0.464	0.825	0.811
	V/C (μm/μm)	13.04	13.63	12.36	14.35	0.62	0.989	0.334	0.597
Jejunum	VH (μm)	814.13	883.12	715.60	861.82	28.00	0.281	0.059	0.485
	CD (μm)	65.81	68.59	68.64	66.80	1.35	0.856	0.869	0.426
	V/C (μm/μm)	12.33	12.87	10.44	12.83	0.38	0.195	0.052	0.209
Ileum	VH (μm)	668.47	696.48	597.15	628.85	18.96	0.079	0.440	0.962
	CD (μm)	80.93	74.12	76.11	85.60	2.89	0.583	0.825	0.184
	V/C (μm/μm)	8.90	9.57	8.34	7.90	0.32	0.093	0.859	0.396
**Day 42**
Duodenum	VH (μm)	1417.00	1369.67	1261.61	1320.79	46.49	0.298	0.951	0.584
	CD (μm)	139.15	113.19	108.09	114.06	7.08	0.298	0.489	0.272
	V/C (μm/μm)	12.01	13.90	12.91	12.28	0.70	0.809	0.672	0.401
Jejunum	VH (μm)	962.97	915.73	896.66	940.08	27.25	0.715	0.973	0.431
	CD (μm)	93.60	98.79	99.91	116.26	3.47	0.082	0.114	0.404
	V/C (μm/μm)	9.45	9.87	9.72	8.73	0.31	0.501	0.654	0.277
Ileum	VH (μm)	687.38	655.45	630.79	600.11	25.53	0.298	0.558	0.991
	CD (μm)	80.06	81.49	87.07	90.84	2.50	0.116	0.612	0.818
	V/C (μm/μm)	8.90	8.41	7.61	6.96	0.31	0.028	0.346	0.895

^a^ Each mean represents 10 replications with 2 broilers per replication. CON, control diet; AFB_1_, 500 μg/kg aflatoxin B_1_ of feed; TA, 250 mg/kg tannic acid of feed; TA + AFB_1_, 250 mg/kg TA + 500 μg/kg AFB_1_.

^b^ CD, crypt depth; V/C, villus height and crypt depth ratio; VH, villus height.

### Antioxidant capacity

The results of the antioxidant capacity in the plasma are shown in Table 5, AFB_1_ challenge decreased plasma CAT and GSH-Px activities on day 21 (*P* < 0.05). Compared with the diet without TA, TA supplementation increased CAT activity in plasma on day 42 (*P* < 0.05). The AFB_1_ and TA exhibited interactive effects on the T-AOC, GST, GSH-Px, and MDA (*P* < 0.05). Compared with the CON, dietary expose to AFB_1_ decreased the T-AOC, GSH-Px, and GST activities on days 21 and 42, and increased the MDA content on day 42, respectively (*P* < 0.05). The addition of TA to AFB_1_ contaminated diet significantly improved the CAT, GSH-Px, and, GST activities, and decreased the MDA content on day 42 (*P* < 0.05).

**Table 5:** Effects of tannic acid on plasma antioxidant capacity of broilers challenged with AFB${}_{1}^{\text{a}}$.

**Items^b^**	**CON**	**TA**	**AFB_1_**	**TA + AFB_1_**	**SEM**	***P-*value**		
						**AFB_1_**	**TA**	**AFB_1_ × TA**
**Day 21**
T-AOC (mmol/L)	0.52^a^	0.48^ab^	0.42^b^	0.49^ab^	0.01	0.103	0.723	0.034
CAT (U/mL)	3.35	3.60	2.64	2.85	0.12	0.002	0.304	0.936
GST (U/mL)	19.22	20.32	18.44	20.74	0.32	0.760	0.006	0.312
GSH-Px (U/mL)	1624.26	1698.25	1535.91	1550.99	20.09	0.002	0.222	0.417
T-SOD (U/mL)	105.51	105.02	99.98	103.11	1.25	0.147	0.603	0.475
MDA (nmol/mL)	4.22	4.05	4.52	4.27	0.10	0.198	0.293	0.865
**Day 42**
T-AOC (mmol/L)	0.43	0.40	0.43	0.46	0.01	0.368	0.831	0.351
CAT (U/mL)	3.82	3.89	3.69	3.99	0.04	0.826	0.014	0.118
GST (U/mL)	20.85^a^	21.31^a^	17.29^b^	20.62^a^	0.37	0.001	0.002	0.015
GSH-Px (U/mL)	1782.43^a^	1888.16^a^	1559.46^b^	1894.93^a^	31.21	0.026	<0.001	0.019
T-SOD (U/mL)	108.19	107.23	105.91	110.43	1.60	0.889	0.591	0.409
MDA (nmol/mL)	3.34^b^	3.21^b^	3.92^a^	3.16^b^	0.07	0.030	0.001	0.011

^a^ Each mean represents 10 replications with 2 broilers per replication. CON, control diet; AFB_1_, 500 μg/kg aflatoxin B_1_ of feed; TA, 250 mg/kg tannic acid of feed; TA + AFB_1_, 250 mg/kg TA + 500 μg/kg AFB_1_.

^b^ T-SOD, total superoxide dismutase; GSH-Px, glutathione peroxidase; GST, glutathione S-transferase; T-AOC, total antioxidant capacity; MDA, malondialdehyde.

^a, b, c^Means in the same row with no common superscripts differ significantly (*P* < 0.05).

As presented in Table 6, AFB_1_ challenge decreased the GST and T-AOC in the liver on days 21 and 42, as well as GSH-Px and T-SOD activity on day 21 (*P* < 0.05). Broilers fed the TA diet had greater hepatic CAT, GST, and GSH-Px activities on day 21 (*P* < 0.05). Furthermore, on day 21, AFB_1_ and TA showed interactive effects on the T-SOD in the liver (*P* < 0.05).

**Table 6:** Effects of tannic acid on liver antioxidant capacity of broilers challenged with AFB_1_.

**Items**	**CON**	**TA**	**AFB_1_**	**TA + AFB_1_**	**SEM**	***P-*** **value**		
						**AFB_1_**	**TA**	**AFB_1_ × TA**
**Day 21**
T-AOC (nmol/mgprot)	207.24	235.86	184.09	180.90	5.96	<0.001	0.211	0.120
CAT (U/mgprot)	15.28	18.58	15.81	18.66	0.66	0.813	0.021	0.861
GST (U/mgprot)	23.35	26.88	16.69	20.32	0.84	<0.001	0.006	0.969
GSH-Px (U/mgprot)	63.38	69.81	31.68	44.88	3.11	<0.001	0.022	0.413
T-SOD (U/mgprot)	1692.04^b^	1885.59^a^	1443.62^c^	1413.18^c^	38.77	<0.001	0.103	0.027
MDA (nmol/mgprot)	1.71	1.65	1.73	1.58	0.06	0.864	0.404	0.730
**Day 42**
T-AOC (nmol/mgprot)	143.34	147.58	125.06	126.11	3.78	0.008	0.713	0.824
CAT (U/mgprot)	19.32	18.96	19.49	19.84	0.60	0.676	0.998	0.778
GST (U/mgprot)	52.71	54.63	47.56	44.78	1.27	0.002	0.851	0.315
GSH-Px (U/mgprot)	58.87	56.95	51.96	60.32	1.51	0.552	0.282	0.090
T-SOD (U/mgprot)	1766.21	1723.15	1606.53	1802.90	37.69	0.595	0.310	0.117
MDA (nmol/mgprot)	2.01	1.86	2.00	1.70	0.09	0.642	0.202	0.674

^a^Each mean represents 10 replications with 2 broilers per replication. CON, control diet; AFB_1_, 500 μg/kg aflatoxin B_1_ of feed; TA, 250 mg/kg tannic acid of feed; TA + AFB_1_, 250 mg/kg TA + 500 μg/kg AFB_1_.

^b^ T-SOD, total superoxide dismutase; GSH-Px, glutathione peroxidase; GST, glutathione S-transferase; T-AOC, total antioxidant capacity; MDA, malondialdehyde.

^a,b,c^Means in the same row with no common superscripts differ significantly (*P* < 0.05).

In Table 7, AFB_1_ challenge decreased the GST and GSH-Px activities in the jejunum on day 21 (*P* < 0.05). Dietary supplemented with TA increased the T-SOD activity in jejunum on day 42. The MDA content of jejunum was also decreased in the TA treatments compared with other treatments (*P* < 0.05).

**Table 7:** Effects of tannic acid on jejunum antioxidant capacity of broilers challenged with AFB${}_{1}^{\text{a}}$.

**Items^b^**	**CON**	**TA**	**AFB_1_**	**TA + AFB_1_**	**SEM**	***P-*** **value**		
						**AFB_1_**	**TA**	**AFB_1_ × TA**
**Day 21**
CAT (U/mgprot)	7.80	9.85	7.58	7.94	0.48	0.275	0.219	0.386
GST (U/mgprot)	21.87	23.25	21.08	21.06	0.35	0.033	0.321	0.308
GSH-Px (U/mgprot)	20.72	21.19	18.31	19.07	0.44	0.009	0.460	0.860
T-SOD (U/mgprot)	275.90	277.80	266.84	277.97	3.07	0.479	0.301	0.462
MDA (nmol/mgprot)	5.02	3.90	4.94	4.47	0.16	0.407	0.010	0.264
**Day 42**
CAT (U/mgprot)	7.10	8.50	8.50	6.73	0.31	0.072	0.225	0.277
GST (U/mgprot)	24.99	27.75	24.47	27.75	0.88	0.883	0.094	0.883
GSH-Px (U/mgprot)	19.93	21.09	17.89	19.37	0.58	0.112	0.261	0.892
T-SOD (U/mgprot)	300.37	330.85	295.25	329.45	5.49	0.747	0.003	0.854
MDA (nmol/mgprot)	4.37	3.99	4.51	4.21	0.12	0.451	0.166	0.876

^a^ Each mean represents 10 replications with 2 broilers per replication. CON, control diet; AFB_1_, 500 μg/kg aflatoxin B_1_ of feed; TA, 250 mg/kg tannic acid of feed; TA + AFB_1_, 250 mg/kg TA + 500 μg/kg AFB_1_.

^b^ T-SOD, total superoxide dismutase; GSH-Px, glutathione peroxidase; GST, glutathione S-transferase; T-AOC, total antioxidant capacity; MDA, malondialdehyde.

## Discussion

Dietary exposure to AFB_1_ can cause tremendous economic losses by reducing growth performance, feed efficiency, and increasing mortality in the poultry industry (20–24). In our study, we found that the administration of 500 μg/kg AFB_1_ diets increased FCR during days 1–21 in broilers. These results are in alignment with several studies, which demonstrated the detriment of broiler health and performance by feeding diets contaminated with 0.1–1 mg/kg AFB_1_ (25, 26). These adverse effects can be explained as AFB_1_ could inhibit protein synthesis and lipogenesis, reduce the activity of digestive enzymes, and change the energy metabolism of the cell (15, 27). We hypothesized that a commercially relevant concentration of AFB1 (500 μg/kg) during 42 days could decrease the growth rate in broilers. Unfortunately, the ADG and ADFI were not affected by the AFB_1_ challenge in the present study. Slizewska et al. (28) also reported that fed 1 mg/kg AFB_1_ of diet did not affect the ADG and ADFI of broilers. Likewise, Chen et al. (29) and Mesgar et al. (30) noted that feed intake, body weight gain, and feed efficiency were not affected by the 500 and 1,000 μg/kg of AFB_1_. Therefore, the toxic effects of AFB_1_ may be acute or chronic, influenced by the age, dose, diet composition, and duration of exposure (31).

In a previous study, we found that 250 and 500 mg/kg TA increased growth performance of broilers (32). In the contrary, supplementation with 250 mg/kg TA had no beneficial effect on the growth performance of broilers. Similar to the current results, Jamroz et al. (33) found that 250–500 mg/kg sweet chestnut tannin had no effect on performance, whereas 1,000 mg/kg TA reduced the final body weight in broilers. In addition, Choi et al. (34) reported that dietary supplementation of 500–5,000 mg/kg TA linearly decreased body weight of boilers infected with *Eimeria Maxima*. On the contrary, Liu et al. (35) found that 1,000 mg/kg chestnut tannins did not affect the body weight gain and feed intake in broilers. Cengiz et al. (36) also indicated that supplemented with 2,000 mg/kg chestnut tannin in broiler diets did not affect the performance. The dosage effect of TA on the growth performance of broilers seems to be unclear. However, it is reported that high dose of TA has negative effects on the growth of broilers, and biological effects are strongly dose-dependent (37, 38). Redondo et al. (39) hypothesized that the addition of excessive TA to the diet may increase the astringency and bitterness of the feed, thereby reducing the feed intake. Based on the different results, the inconsistency might be attributed to the source of tannic acid, administration dosage, diet composition, and age of the bird (40).

Aflatoxin has been known to mainly accumulated and metabolized in the liver and kidney after absorption, causing impairment of the liver and kidney (41, 42). In the present study, we observed that 500 μg/kg AFB_1_ caused a significant increase in the relative weight of liver and kidney, which is consistent with other studies (43–45). The enlargement of organ weight is attributed to disorders of lipid metabolism, and the inhibition of lipid transportation, leading to lipid deposition, which results in hepatomegaly (15, 46). Many studies describe the role of plant extract could ameliorate the adverse effect of AFB_1_ in broilers (47–49). In our previous study, we also found that the increase in liver and kidney relative weight in the AFB_1_ group was ameliorated by the supplementation of 250 and 500 mg/kg TA (32). Therefore, these results confirmed that TA has a protective effect on the liver and kidney damage caused by AFB_1_.

Intestinal villus height, crypt depth, and villus height/crypt depth ratio are important indexes to evaluate intestinal nutrient digestion and absorption capacity of poultry (50). These parameters especially the villus height/crypt depth ratio was positively related to the absorptive efficiency of the intestine (51). In the present study, intestinal histomorphology result revealed that dietary AFB_1_ exposure decreased the villus height and crypt depth ratio in the ileum of 42-day-old broilers. Similar to the results by Tavangar et al. (22), who reported that 1 mg/kg AFB_1_ decreased small intestine villus height and villus height to crypt depth ratio of broilers. These results showed that AFB_1_ could decrease the capacity of intestinal mucosa to digest and absorb nutrients by depressing intestinal development. Brus et al. (52) found that tannin extract could promote the proliferation of intestinal epithelial cells to promote intestinal development *in vitro*. Therefore, further studies need to be conducted to confirm the positive effect of TA on intestinal morphology in broilers.

It has been demonstrated that AFB_1_ could induce the production of reactive oxygen species (ROS) and oxidative stress, thereby inducing cell and DNA damage (53). The antioxidant system of organism can eliminate the adverse effects of ROS, and the GST, T-SOD, CAT, and GSH-Px are important endogenous antioxidant enzymes, which play a key role in scavenging free radicals and maintaining the intracellular redox equilibrium (15). In the present study, AFB_1_ significantly increased the concentrations of MDA and decreased the antioxidant enzyme activities of T-SOD, GSH-Px, GST, and CAT in the liver, jejunum, and the plasma of broilers when compared with the CON. These results are in consistent with previous studies, which demonstrated that different dosage of AFB_1_ decreased the activity of antioxidant enzymes, increased the lipid peroxidation, and inhibited the antioxidant capacity of broilers (14, 15, 54, 55). Recently, researchers have been interested in the usage of antioxidants to counter the toxic effects of aflatoxins (56). Our present results confirmed that 250 mg/kg TA could enhance antioxidative capacity, and alleviate the adverse effects of AFB_1_ on oxidative stress in the liver, jejunum, and plasma, which is similar to previous studies (57–59). Consequently, these results indicated that TA could play an important role in preventing the AFB_1_-induced oxidative damage in broilers.

## Conclusion

In conclusion, supplementation with 250 mg/kg TA could alleviate the oxidative damage, and prevent the enlargement of liver in broilers dietary challenge with 500 μg/kg AFB1. Therefore, Chinese gallnut TA may be used as a feed additive in the prevention of aflatoxicosis and improve the health of poultry.

## Data availability statement

The original contributions presented in the study are included in the article/supplementary material, further inquiries can be directed to the corresponding author/s.

## Ethics statement

The animal study was reviewed and approved by Institutional Animal Care and Use Committee of Wuhan Polytechnic University (Number: 20161121).

## Author contributions

ZZ and BD conceived and designed the experiment. YX, JC, SG, SW, ZL, and LL performed the experiment. YX, ZL, and YQ analyzed the data. YX and ZZ wrote the manuscript. All authors read and approved the final manuscript.

## Funding

This research was financially supported by the Key Projects of Hubei Province (No. 2022BBA0014) and the Research and Innovation Initiatives of Wuhan Polytechnic University (No. 2022RZ068).

## Conflict of interest

The authors declare that the research was conducted in the absence of any commercial or financial relationships that could be construed as a potential conflict of interest.

## Publisher's note

All claims expressed in this article are solely those of the authors and do not necessarily represent those of their affiliated organizations, or those of the publisher, the editors and the reviewers. Any product that may be evaluated in this article, or claim that may be made by its manufacturer, is not guaranteed or endorsed by the publisher.
